# Hydrogen phosphate selectively induces MDA MB 231 triple negative breast cancer cell death in vitro

**DOI:** 10.1038/s41598-022-09299-2

**Published:** 2022-03-29

**Authors:** Aya Shanti, Kenana Al Adem, Cesare Stefanini, Sungmun Lee

**Affiliations:** 1 Department of Biomedical Engineering, Healthcare Engineering Innovation Center, Khalifa University of Science and Technology, 127788 Abu Dhabi, United Arab Emirates; 2 Khalifa University’s Center for Biotechnology, Khalifa University of Science and Technology, 127788 Abu Dhabi, United Arab Emirates

**Keywords:** Breast cancer, Cancer metabolism, Cancer therapy

## Abstract

Phosphate ions are the most abundant anions inside the cells, and they are increasingly gaining attention as key modulators of cellular function and gene expression. However, little is known about the effect of inorganic phosphate ions on cancer cells, particularly breast cancer cells. Here, we investigated the toxicity of different phosphate compounds to triple-negative human breast cancer cells, particularly, MDA-MB-231, and compared it to that of human monocytes, THP-1. We found that, unlike dihydrogen phosphate (H_2_PO_4_^−^), hydrogen phosphate (HPO_4_^2−^) at 20 mM or lower concentrations induced breast cancer cell death more than immune cell death, mainly via apoptosis. We correlate this effect to the fact that phosphate in the form of HPO_4_^2−^ raises pH levels to alkaline levels which are not optimum for transport of phosphate into cancer cells. The results in this study highlight the importance of further exploring hydrogen phosphate (HPO_4_^2−^) as a potential therapeutic for the treatment of breast cancer.

## Introduction

Inorganic phosphate (P_i_) is a vital nutrient for all living things. It plays a crucial role in various physiological processes such as energy metabolism, lipid biosynthesis, nucleic acid biosynthesis, cellular differentiation, cellular repair and cellular signaling^1,2^. The main source of P_i_ intake for humans is the diet, where P_i_ exists in one of three anionic forms; HPO_4_^2−^, H_2_PO_4_^−^ or H_3_PO_4_^2^. In the body, serum P_i_ levels are precisely maintained within a specific range via the interplay between several organs including intestine, kidney, parathyroid gland and bones. Similarly, extracellular P_i_ levels are maintained within a narrow range; between 0.7 and 1.55 mM^2–4^.

Many studies have proposed the existence of a phosphate sensing mechanism in the body capable of detecting both serum and extracellular phosphate fluctuations and subsequently relaying this information to the cells, the local environment and/or the whole body^5–7^. Interestingly, several findings have revealed that extracellular phosphate in itself carries out this function by acting as a signaling molecule^6,8–12^. The interior environment of the cell is electronegative compared to the exterior one, thus, the transport of P_i_ into the cell does not happen by simple diffusion, but instead is mediated by Na^+^-coupled P_i_ cotransporters, which are highly regulated proteins^4,5,13^. Furthermore, P_i_ can initiate signal transduction pathways, alter gene expression and regulate diverse cellular functions^7,9,14,15^. All these evidence serve to pinpoint the importance of P_i_ in the body and to highlight the significance of regulating P_i_ levels.

Recently, high P_i_ intake and high P_i_ serum levels were associated with higher morbidity and mortality rates^16,17^. In fact, high serum concentrations are associated with kidney disease, perturbed brain growth, vascular calcification and cardiovascular events^16–19^. However, the mechanisms by which high P_i_ concentrations are linked to tissue damage are not completely understood. Shuto et.al showed that P_i_ acutely impairs endothelial function by increasing production of reactive oxygen species and decreasing production of nitric oxide^20^. On the other hand, how phosphate affects diseased cells, particularly cancer cells, is only minimally explored^21,22^. For instance, Spina et.al demonstrated that P_i_ inhibits proliferation of human osteosarcoma U2OS cells as well as MDA-MB-231 cells but did not explore its effect on normal, healthy cells^14,23,24^.

In this study, we investigated the toxicity of different phosphate compounds to triple-negative human breast cancer cells (MDA-MB-231) and to human monocytes (THP-1). We demonstrated that, unlike phosphate in the form of dihydrogen phosphate (H_2_PO_4_^−^), hydrogen phosphate (HPO_4_^2−^) induces breast cancer cell death but not immune cell death. In addition, we showed that hydrogen phosphate induces MDA-MB-231 cell death particularly via apoptosis. We attribute this effect to the fact that phosphate in the form of HPO_4_^2−^ raises pH levels to alkaline levels which are not optimum for phosphate transport into cancer cells. Taken together, these results indicate the significance of further exploring HPO_4_^2−^ as potential therapeutic for the treatment of breast cancer.

## Results

### Cytotoxicity of different phosphate compounds

**Figure 1:**
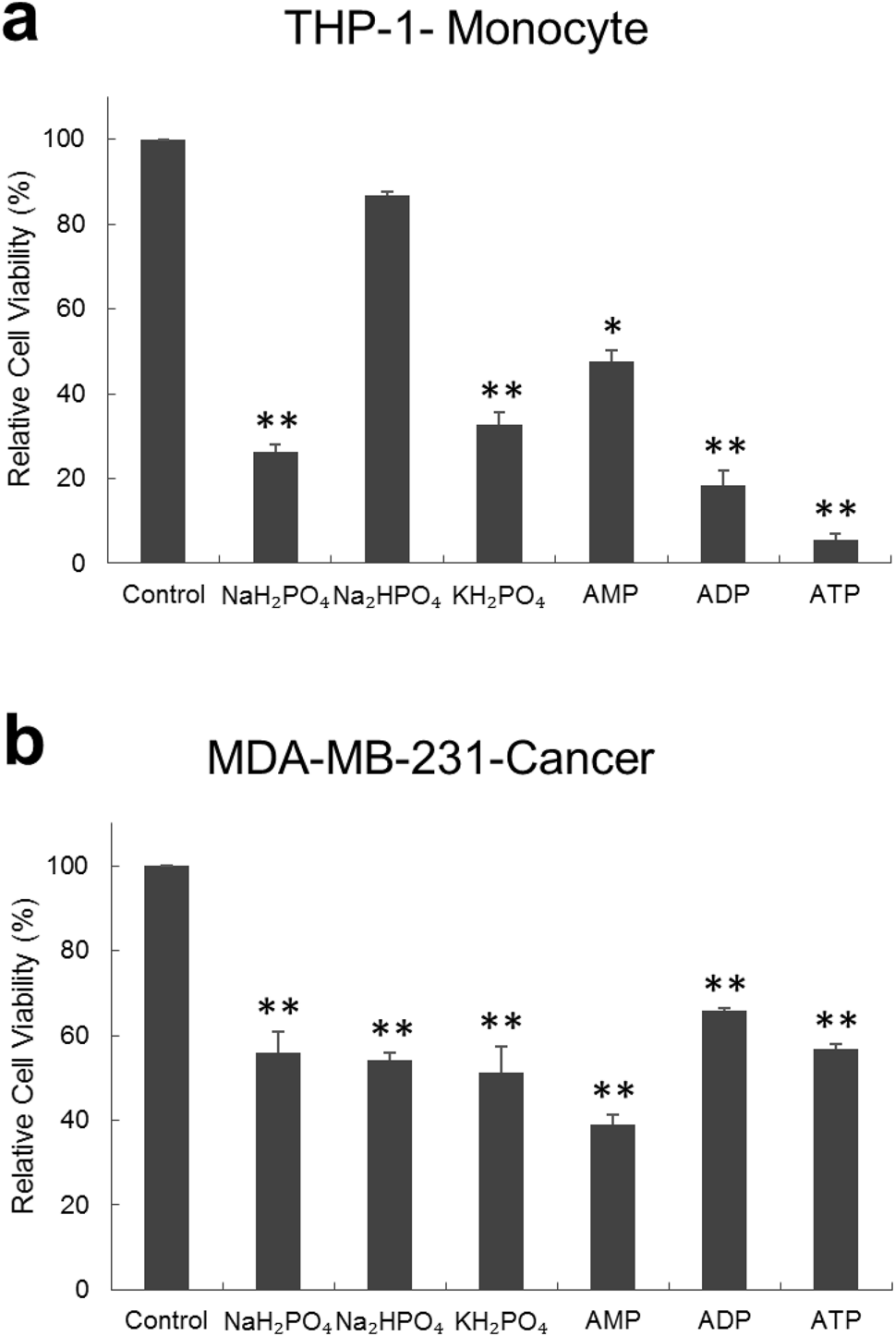
Toxicity of different phosphate compounds to (**a**) THP-1, human monocyte cell line, and (**b**) MDA-MB-231, human breast cancer cell line, evaluated after 48 h of incubation with the phosphate compound. All phosphate-containing compounds reduced viability of both cell types significantly except Na_2_HPO_4_, which only reduced the viability of MDA-MB-231 cells but not that of THP-1 cells. Experiment was done in triplicate and the sample number for each replicate is 3.

In order to assess the effect of different phosphate compounds on the cell viability of MDA-MB-231 and THP-1 cells, three inorganic phosphates (NaH_2_PO_4_, Na_2_HPO_4_, and KH_2_PO_4_) and three organic phosphates (adenosine triphosphate (ATP), adenosine diphosphate (ADP) and adenosine monophosphate (AMP)) were tested. Both MDA-MB-231 and THP-1 cells were incubated with 20 mM of NaH_2_PO_4_, Na_2_HPO_4_, KH_2_PO_4_, ATP, ADP and AMP for 48 h, then assessed for their viability via MTT assay. As shown in Fig. 1, almost all phosphate-containing compounds were toxic (viability < 80%) to both cell types and their toxicity was more pronounced on THP-1 cells than it was on MDA-MB-231 cells. Interestingly, only Na_2_HPO_4_ was more toxic to MDA-MB-231 cells than it was to THP-1 cells. In particular, the viability of MDA-MB-231 cells incubated with 20 mM Na_2_HPO_4_ was 37.5% lower than that of THP-1 (relative viability of MDA-MB-231 was 54.1% while that of THP-1 was 86.6%). This indicates that the toxicity of Na_2_HPO_4_ is selective to the cell type.

**Figure 2:**
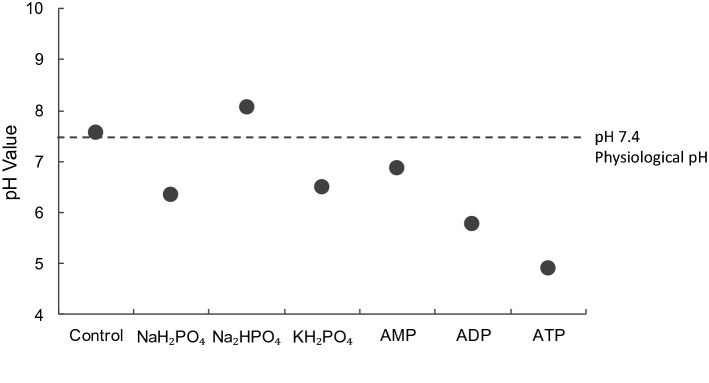
Measured pH values for different phosphate compounds prepared in cell culture media. All compounds produced acidic solutions except Na_2_HPO_4_ which produced a basic solution.

In order to understand the difference in the effect of the various phosphate compounds on the two cell types, the pH of the different phosphate solutions was measured. In Fig. 2, all phosphate compounds produced acidic solutions except Na_2_HPO_4_ which produced a basic one. In supporting Fig. 1, the cytotoxicity of the different phosphate compounds was compared to controls at suitable pH (i.e. to cells incubated in media with no phosphate compound but having their media pH adjusted by either HCl or NaOH to a level nearly equal to that containing phosphate compound). The figure shows that pH is a factor contributing to the toxicity of Na_2_HPO_4_ to MDA-MB-231 cells but is not the only one. This, as well as the fact that Na_2_HPO_4_ is the only compound containing phosphate in the form of HPO_4_^2−^, were a drive to further explore Na_2_HPO_4_ compound.

In the medium, inorganic phosphate compounds are dissociated into ions.

$$ {\text{NaH}}_{{2}} {\text{PO}}_{{4}} \leftrightarrow {\text{Na}}^{ + } + {\text{H}}_{{2}} {\text{PO}}_{{4}}{^{ - }} $$

$$ {\text{Na}}_{{2}} {\text{HPO}}_{{4}} \leftrightarrow {\text{2Na}}^{ + } + {\text{HPO}}_{{4}}{^{{{2} - }}} $$

$$ {\text{KH}}_{{2}} {\text{PO}}_{{4}} \leftrightarrow {\text{K}}^{ + } + {\text{H}}_{{2}} {\text{PO}}_{{4}}{^{ - }} $$

Na_2_HPO_4_ produces phosphate ion in the form of HPO_4_^2−^, i.e. hydrogen phosphate.

### Effect of hydrogen phosphate (HPO_4_^2−^) on MDA-MB-231 and THP-1 cell viability

**Figure 3:**
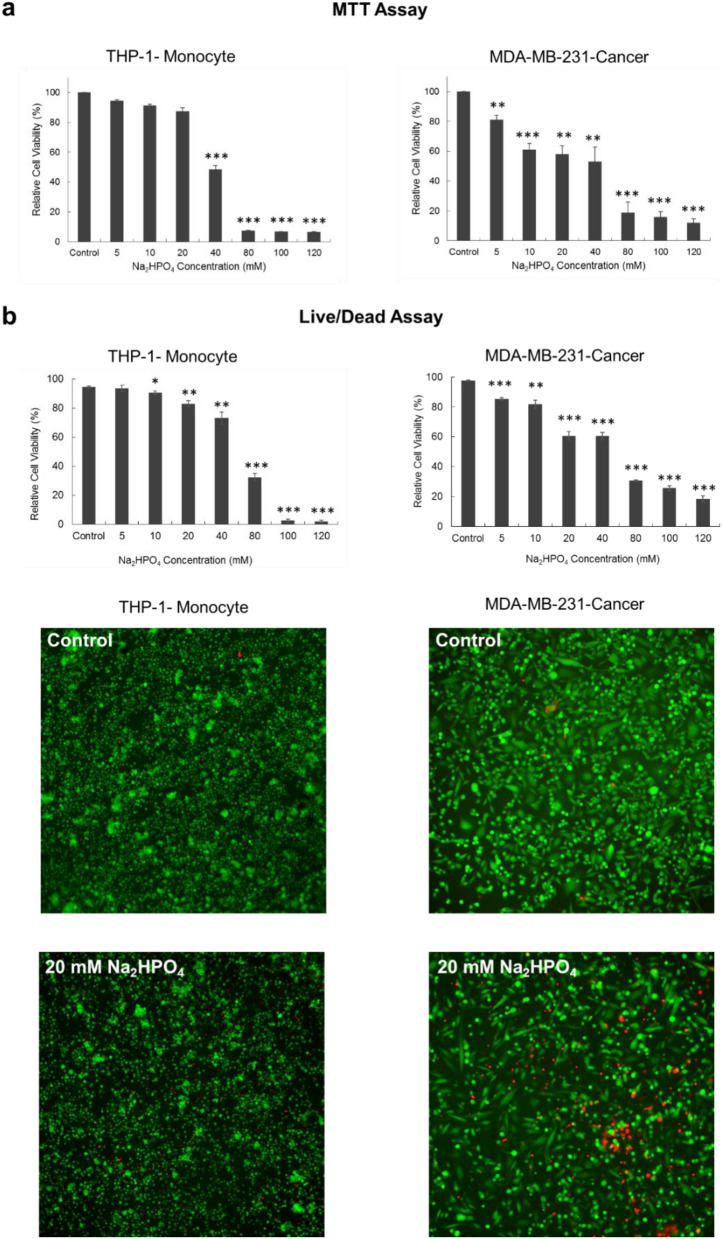
Toxicity of Sodium Phosphate Dibasic (Na_2_HPO_4_) to THP-1, human monocyte cell line and MDA-MB-231, human breast cancer cell line, evaluated via (**a**) MTT assay and (**b**) live/dead assay after 48 h of incubation with the compound. At low concentrations (< or = 20 mM), Na_2_HPO_4_ is more toxic to MDA-MB-231 cells than it is to THP-1 cells. At high concentrations (> 20 mM), Na_2_HPO_4_ is toxic to both cell types. For fluorescence images, live cells are stained in green while dead cells are stained in red. Experiments were done in triplicate and the sample number for each replicate is 3.

In order to further examine the effect of hydrogen phosphate (HPO_4_^2−^) on viability of MDA-MB-231 and THP-1 cells, cells were incubated with different concentrations of Na_2_HPO_4_ for 48 h then assessed for their viability via MTT or live/dead assay. Figure 3 (both MTT and live/dead) demonstrates that, at low concentrations, HPO_4_^2−^ was more toxic to MDA-MB-231 cells than it was to THP-1 cells. Interestingly, for MDA-MB-231, even low concentrations (< 20 mM) caused a significant decrease in cell viability. For example, 5 mM Na_2_HPO_4_ reduced the viability of MDA-MB-231 by almost 20%. However, for THP-1 cells, there was no significant decrease in the viability of cells incubated with 5 mM, 10 mM and 20 mM Na_2_HPO_4_ compared to control cells. Only concentrations above 20 mM produced a significant decrease in viability. Figure 3b (bottom) shows fluorescence images of both THP-1 and MDA-MB-231 cells incubated in 20 mM Na_2_HPO_4_ for 48 h and stained with live/dead stains. Live cells are stained green and dead cells are stained red. Supplementary Fig. 2 compares the effect of different concentrations of sodium phosphate monobasic and sodium phosphate dibasic on both THP-1 cells and MDA-MB-231 cells.

**Figure 4:**
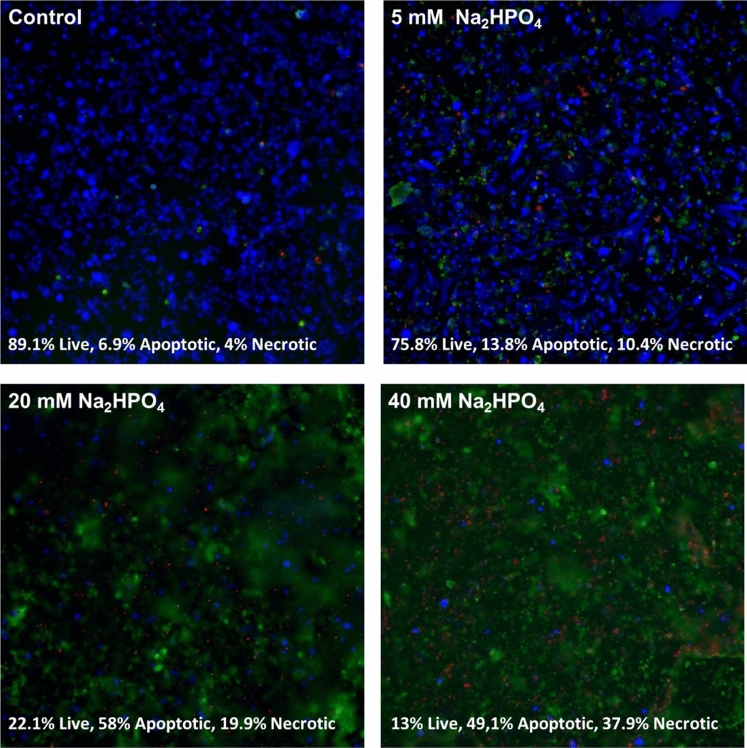
Mechanism of cell death induced by Sodium Phosphate Dibasic on MDA-MB-231 determined after 48 h of incubation with the phosphate compound. Live cells are shown in blue, apoptotic cells are shown in green and necrotic cells are shown in red. Sodium Phosphate Dibasic induces both necrosis and apoptosis, with apoptosis being more pronounced.

In order to examine the type of death induced by Na_2_HPO_4_ on MDA-MB-231 cells, cells were treated with various concentrations of Na_2_HPO_4_ for 48 h then stained by apoptosis/necrosis dyes. As seen in Fig. 4, results indicate that Na_2_HPO_4_ induces both necrosis and apoptosis, with apoptosis being more pronounced (particularly at concentrations of 20 mM and 40 mM).

**Figure 5:**
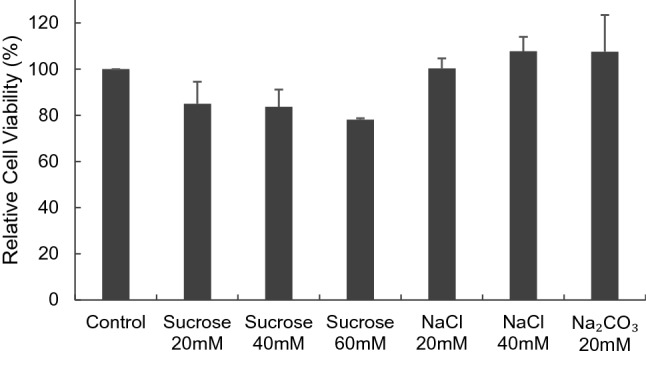
Effect of Sodium and osmotic pressure on viability of MDA-MB-231, human breast cancer cell line. In order to confirm that the observed toxicity of Na_2_HPO_4_ on MDA-MB-231 was induced by phosphate and not by sodium in NaHPO_4_ or by simple osmotic pressure, the toxicity of NaCl, sucrose and sodium bicarbonate on MDA-MB-231 was assessed. None of the compounds caused a significant decrease in the viability of cells. Experiment was done in triplicate and the sample number for each replicate is 3.

In order to confirm that the observed toxicity of Na_2_HPO_4_ on MDA-MB-231 was induced by phosphate and not by sodium in Na_2_HPO_4_ or by simple osmotic pressure, the toxicity of NaCl, sucrose and sodium bicarbonate (NaHCO_3_) on MDA-MB-231 was assessed. In Fig. 5, none of the compounds caused a significant decrease in the viability of cells which indicates that the phosphate is the probable cause for the induced toxicity observed.

**Figure 6:**
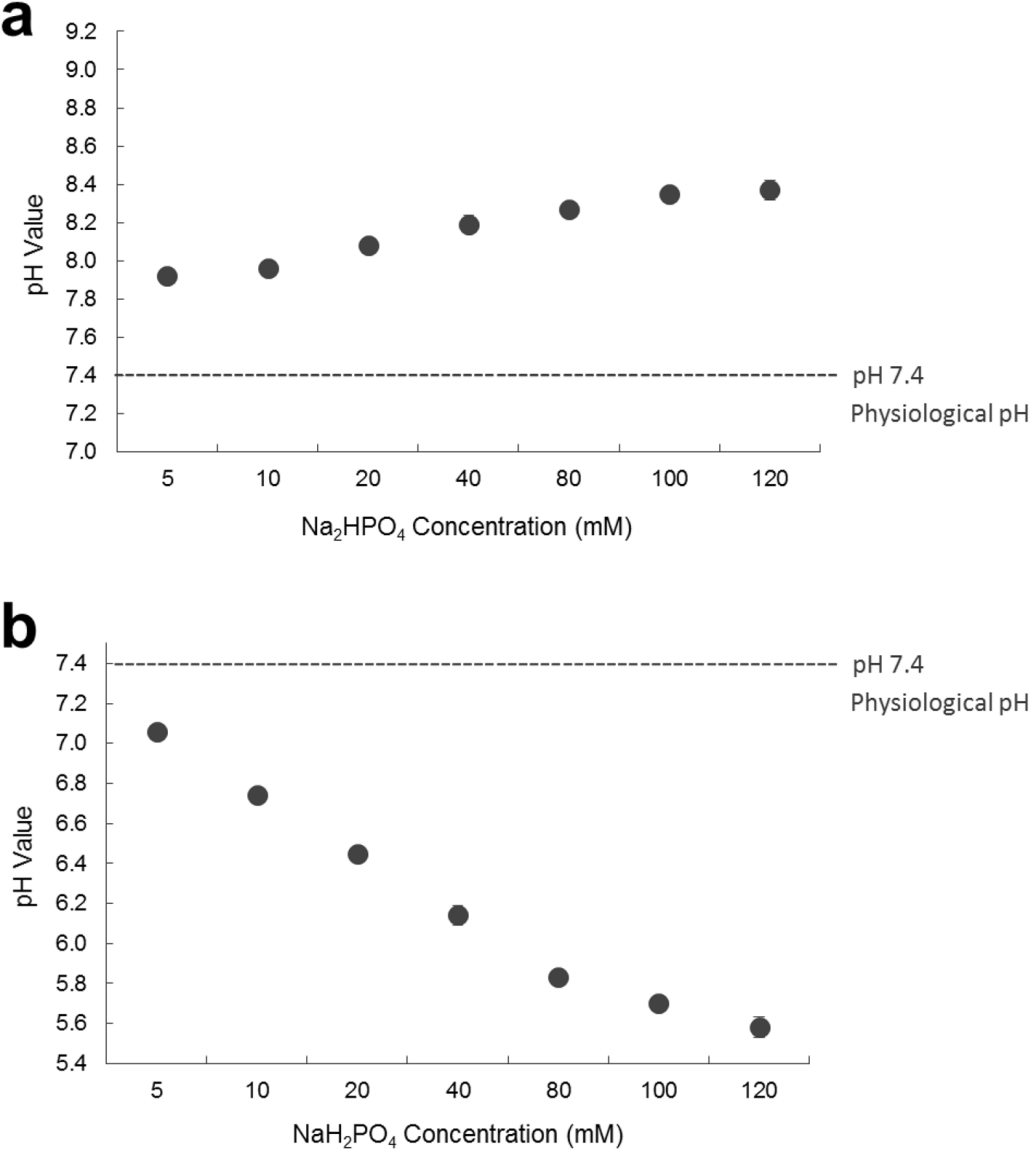
Measured pH values for different concentrations of (**a**) Sodium phosphate dibasic and (**b**) Sodium phosphate monobasic prepared in cell culture media. As the concentration of HPO_4_^2−^ increases, the pH of the solutions increases. As the concentration of H_2_PO_4_^−^ increases, the pH of the solutions decreases.

In order to explore the possible mechanisms of action in which HPO_4_^2−^ induces its toxicity, the effect of HPO_4_^2−^ on pH of the media was assessed. For that, phosphate solutions at different concentrations of Na_2_HPO_4_ (5, 10, 20, 40, 80, 100 and 120 mM) were prepared in media then their pH measured. As seen in Fig. 6a, results indicate that as the concentration of HPO_4_^2−^ increases, the pH of the solutions increases. These results are in contrast to those of H_2_PO_4_^−^, Fig. 6b, in which the pH decreases with increasing NaH_2_PO_4_ concentration.

### Tolerance of MDA-MB-231 and THP-1 cells to different pH levels

**Figure 7:**
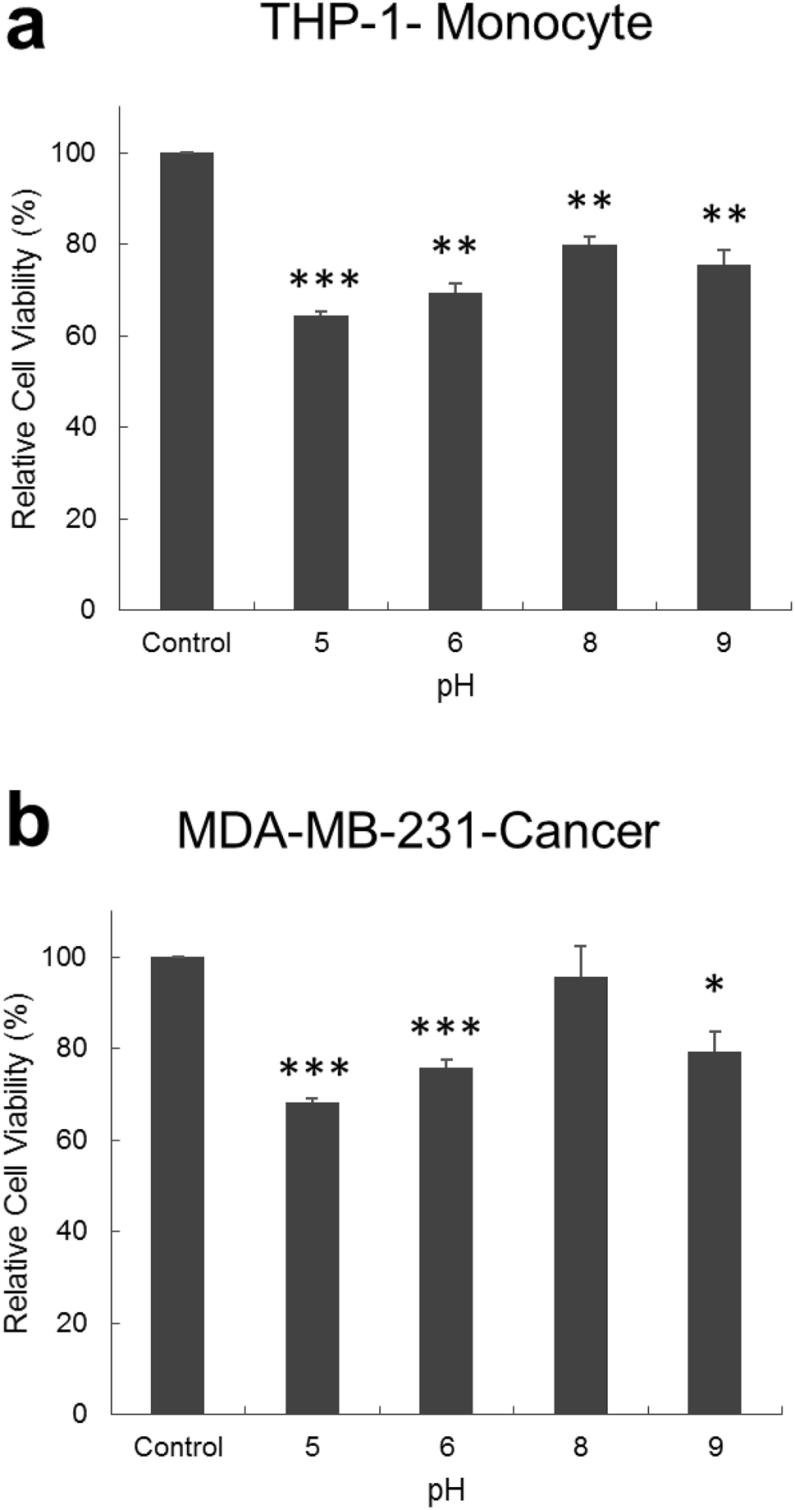
Viability of (**a**) THP-1, human monocyte cell line, and (**b**) MDA-MB-231, human breast cancer cell line, at different pH levels evaluated after 48 h of incubation. Acidic conditions caused a significant decrease in the viability of both cell types. For basic conditions, at pH 8, the viability of THP-1 cells decreased significantly but that of MDA-MB-231 did not. At pH 9, viability of both cell types decreased significantly. Experiment was done in triplicate and the sample number for each replicate is 3.

As HPO_4_^2−^ was found to alter the pH of the media, the difference in the tolerance of MDA-MB-231 and THP-1 cells to different pH levels was examined. For this, cells were incubated in media solutions at pH values equal to 5, 6, 7, 8 and 9 for 48 h then their viability determined using MTT assay. As seen in Fig. 7, results demonstrated that both cell types were more tolerant to basic conditions than they were to acidic conditions, and that MDA-MB-231 cells were slightly more tolerant to pH fluctuations than THP-1 cells were.

### Effect of sodium phosphate dibasic (Na_2_HPO_4_) at physiological pH

If MDA-MB-231 cells are more tolerant to pH fluctuations than THP-1 cells are, there should be another reason why Na_2_HPO_4_ is more toxic to MDA-MB-231 than it is to THP-1 cells. Our hypothesis is that this toxicity is related to the way phosphate is transported into cancer cells, particularly breast cancer cells. Several studies showed that breast cancer cells have high levels of Pi transporters compared to normal tissue^25–27^. These transporters, according to Forster et al., Russo-Abrahão et.al and Takeda, are sodium dependent and bind with high affinity to inorganic phosphate^1,28,29^. Interestingly, they have high affinity for P_i_ in its diprotic form (H_2_PO_4_^−^), which exists at physiological pH, and low affinity for P_i_ in its monoprotic form (HPO_4_^2−^), which exists at alkaline pH ^1^. Since triple negative breast cancer cells are known to require elevated amounts of phosphate to meet their metabolic demands, failure to efficiently transport phosphate into cancer cells alters their functioning and probably leads to cellular death^30,31^. This study demonstrates that phosphate in its monoprotic form (HPO_4_^2−^) causes significant death in MDA-MB-231 cells but not in THP-1 cells, unlike phosphate in its diprotic form.

**Figure 8:**
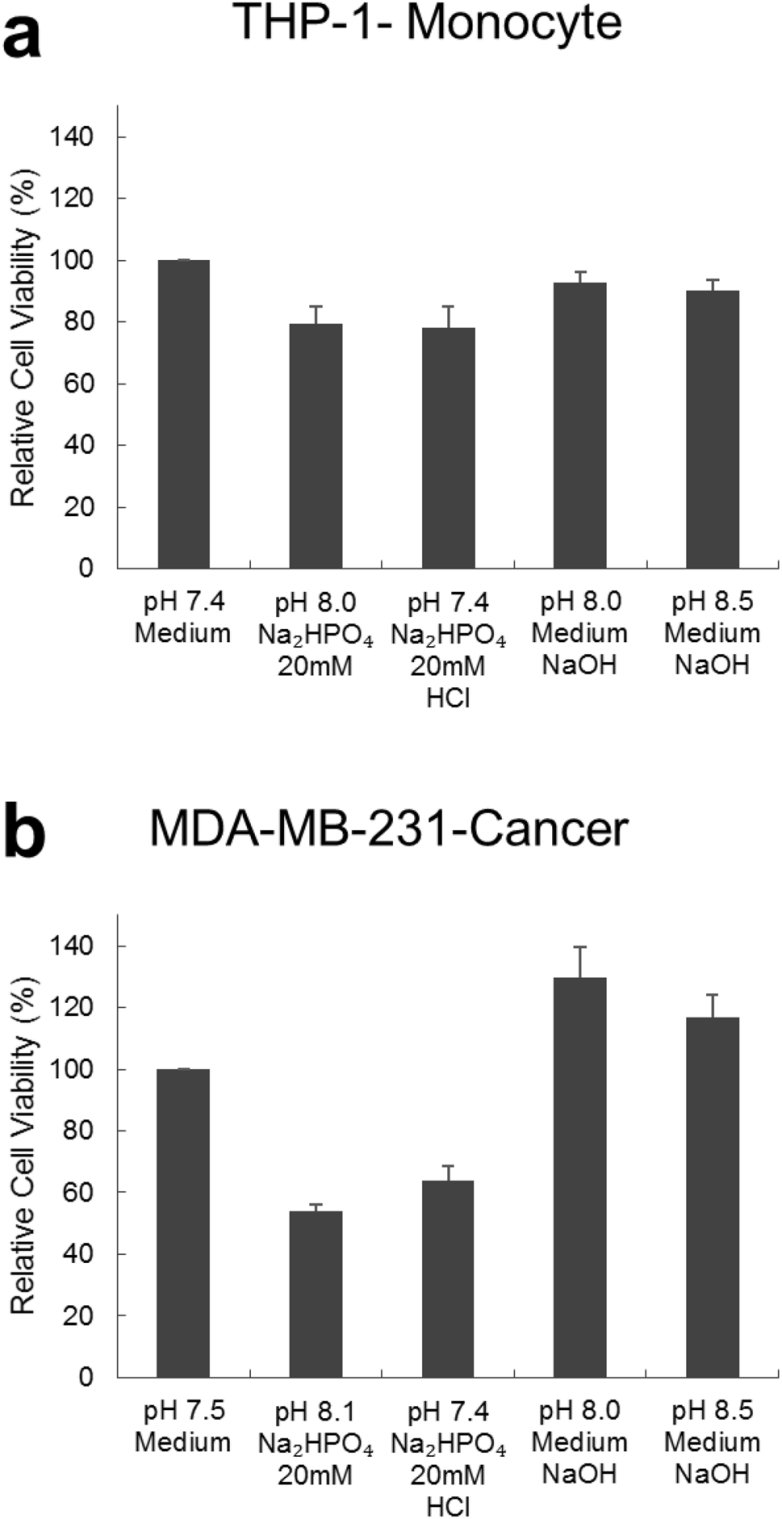
Viability of (**a**) THP-1, human monocyte cell line, and (**b**) MDA-MB-231, breast cancer cell line, after adding Sodium Phosphate Dibasic (Na_2_HPO_4_) and adjusting pH to physiological one, evaluated after 48 h of incubation with the compound. Adjusting the pH to physiological range (7.4–7.5) after adding Na_2_HPO_4_ increased the viability of cells by 18% (approaching significance). Media only and media with NaOH are controls. Experiment was done in triplicate and the sample number for each replicate is 3.

Since phosphate in general is a tryptotic acid and, thus, has different physiological forms according to the pH range in which it is found, the effect of adding Na_2_HPO_4_ to MDA-MB-231 and THP-1 cells and then adjusting the pH to a physiological one (pH = 7.4) was also explored^32^. As seen in Fig. 8, results demonstrate that adjusting the pH to physiological range after adding HPO_4_^2−^ slightly enhanced viability of MDA-MB-231 cells to near significant values but had no effect on viability of THP-1 cells. The level of enhancement in viability was also pH dependent. These results support the hypothesis that cancer cells seem to favor diprotic form of phosphate than monoprotic one.

## Discussion

In this study, we demonstrated the potential of HPO_4_^2−^ to contribute to the treatment of triple negative breast cancer cells. This is quite significant as there is no effective therapy for triple-negative breast cancer to date and it continues to acclaim the lives of its patients^33,34^. P_i_ is emerging as a crucial signaling molecule capable of altering signal transduction pathways, gene expression, and protein abundance in many cell types^9,35–37^. Interestingly, the effect of phosphate on cells is not universal; it varies according to the cellular type and the cellular background. In this study, we showed via MTT and live/dead assays that phosphate, in the form of HPO_4_^2−^, induces cell death in a specific type of triple negative breast cancer cells, namely MDA-MB-231 cells, but not in monocytes (THP-1 cells). This is done via both apoptosis and necrosis with apoptosis being more pronounced. In other studies, P_i_ was shown to increase cell proliferation in some cell types, such as preosteoblastic cells, lung cells, and epidermal JB6 cells while decreasing proliferation in other cell types such as human osteosarcoma U2OS cells and MDA-MB-231 cells^3,19,23,23,35,38–41^. In addition, P_i_ was shown to induce apoptosis in MO6-G3 odontoblast-like cells^42^. Interestingly, Spina et.al showed that Pi inhibits proliferation in MDA-MB-231 cells but not in MCF-7 breast cancer cells, which are not “triple negative” and express estrogen and progesterone receptors^14^. Taken together, these results strongly suggest that Pi has a discrete effect on cells depending on their type and their background.

**Figure 9:**
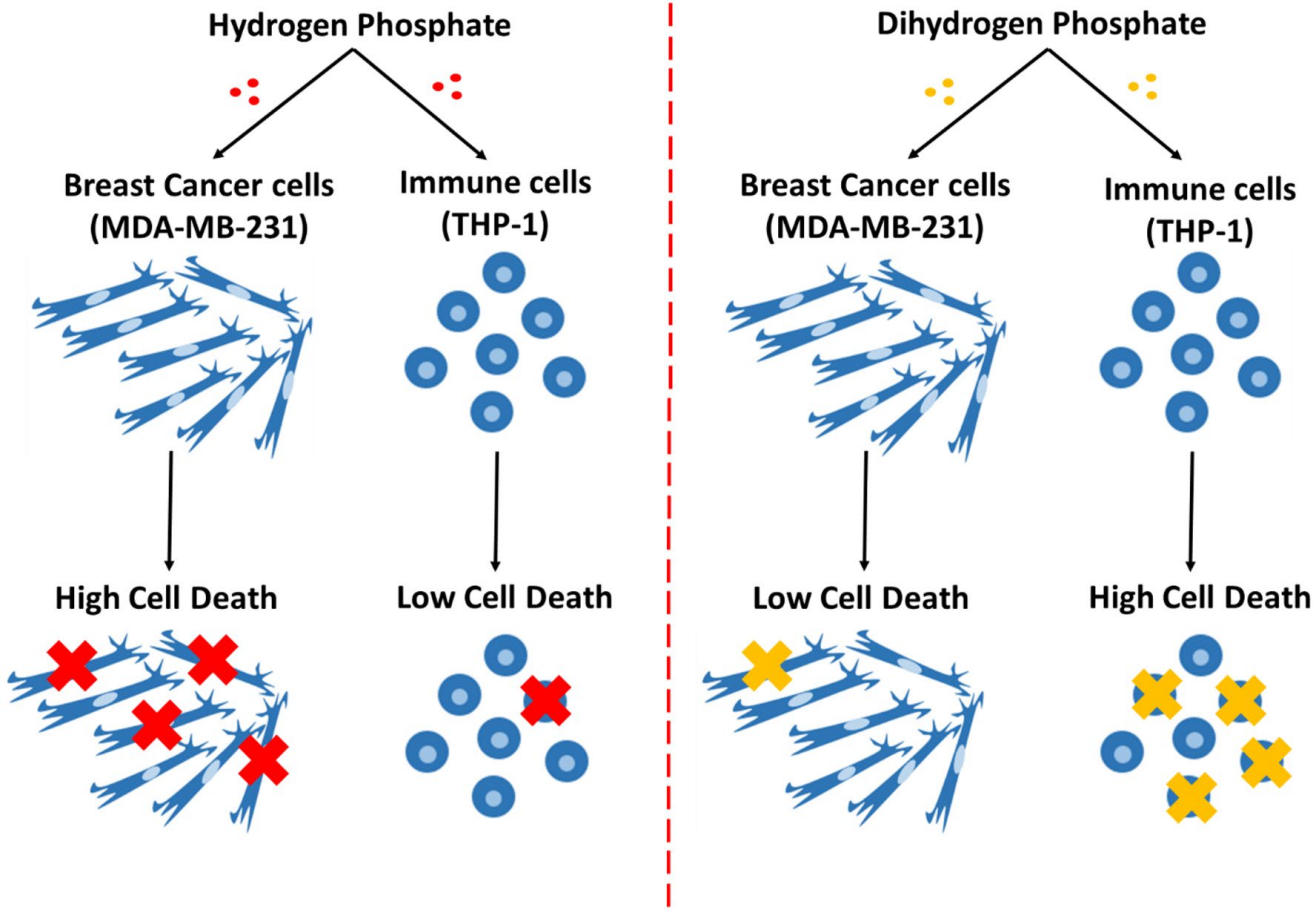
Schematic diagram—effect of hydrogen phosphate vs. dihydrogen phosphate on the cell viability of MDA-MB-231 and THP-1 cells. Hydrogen phosphate causes significant death in MDA-MB-231 cells but not in THP-1 cells, unlike dihydrogen phosphate.

In this study, we also show that the specific type of phosphate available to the cells has a great influence on its response to it. In particular, hydrogen phosphate causes significant death in MDA-MB-231 cells but not in THP-1 cells, unlike dihydrogen phosphate. Figure 9 is a schematic showing the observed effect of hydrogen phosphate and dihydrogen phosphate on the viability of both MDA-231 cells and THP-1 cells. We hypothesize that this effect is related to the fact that MDA-MB-231 cells require high amounts of phosphate to meet their metabolic needs but are unable to attain it due to the inability of phosphate transporters to carry phosphate into the cells when it is in the form of HPO_4_^2−^^43^. Studies show that cancer cells depend on several transporters to transport phosphate into the cells, but the key ones are sodium dependent transporters and hydrogen dependent transporters, both of which are highly overexpressed in MDA-MB-231 cells^1,2,28,29^. Interestingly, sodium dependent phosphate transporters have higher affinity for dihydrogen phosphate (H_2_PO_4_^−^) than they do to hydrogen phosphate (HPO_4_^2−^)^1^. In addition, hydrogen dependent transporters tend to transport phosphate more efficiently in acidic conditions than they do in basic conditions due to the greater availability of protons (H+) in acidic environments compared to basic environments^2^. These findings are, in fact, in consistence with the results obtained from this study in which hydrogen phosphate was more toxic to MDA-MB-231 cells than dihydrogen phosphate.

Finally, we suggest that hydrogen phosphate is a suitable candidate for further investigation as a therapeutic for the treatment of triple negative breast cancer, due to its selective ability to induce cell death in cancer cells but not in immune cells. This is quite crucial as effective cancer therapeutics require high antitumor activity and minimal toxicity to normal tissues. We emphasize here that the effect of phosphate on cells is dependent on their type. In this study, phosphate, in the form of HPO_4_^2−^ was shown to induce cell death in MDA-MB-231 cells but may not have the same effect in other breast cancer cell types. Therefore, future work would include examining the effect of hydrogen phosphate on various cell types (non-cancerous cells and non-triple negative breast cancer cells) and at various concentrations and studying the effect of hydrogen phosphate using in vivo models.

## Materials and methods

### Cell culture

MDA-MB-231 (ATCC, Manassas, VA, USA) cells were cultured in Dulbecco's Modified Eagle Medium (DMEM) containing 4.5 g/L d-glucose, 0.584 g/L l-Glutamine and 0.11 g/L Sodium Pyruvate (Gibco, Fisher Scientific, Waltham, MA, USA). The media was supplemented with 10% Fetal Bovine Serum (FBS) (Gibco) and 1% penicillin–streptomycin (Gibco). The cells were incubated at 37 °C and 5% CO_2_.

THP-1 (ATCC) cells were cultured in Roswell Park Memorial Institute (RPMI) 1640 medium containing 4.5 g/L d-glucose, 0.3 g/L l-glutamine, and 0.11 g/L sodium pyruvate (Gibco). The media was supplemented with 10% fetal bovine serum (FBS) and 1% penicillin–streptomycin (Gibco).

All cells were incubated at 37 °C and 5% CO_2_.

### Cytotoxicity of different phosphate compounds

To assess the toxicity of different phosphate compounds on MDA-MB-231 cells and on THP-1 cells, an MTT (3-(4,5-dimethylthiazol-2-yl)-2,5-diphenyltetrazolium bromide) (Sigma-Aldrich, St. Louis, MO, USA) reduction assay was performed. In brief, cells were seeded in 96 well plates at a concentration of 6 × 10^5^ cells/mL and volume of 100 µL. Cells were then incubated for 24 h at 37 °C and 5% CO_2_. After 24 h of incubation, 20 mM of NaH_2_PO_4_, KH_2_PO_4_, Na_2_HPO_4_, ATP, ADP and AMP were added to cells while maintaining a total volume of 200 µL for each sample. Cells with 200 µL media and no phosphate were kept as a control. Cells were then incubated for an additional 48 h. After 48 h, 20 µL of MTT were added to the cells and the cells were subsequently incubated for extra 4 h. During this incubation time, yellow MTT solution was reduced to purple formazan in living cells. After 4 h, media containing MTT was removed from all wells and replaced by DMSO. The absorbance of each sample was then measured at 570 nm using the microplate reader (Tecan Trading AG, Zurich, Switzerland). Percentage of viable cells was calculated by comparing the absorbance of the control cells (cells with medium only and no phosphate) to that of cells with the phosphate. Percentage cell viability was calculated as: Percentage cell viability = (Absorbance of cells with phosphate and MTT)/(Absorbance of control cells with MTT but no phosphate) × 100.

### Cytotoxicity of sodium phosphate dibasic

To further examine the effect of phosphate in the form of HPO_4_^2−^ on MDA-MB-231 cells and on THP-1 cells, cells were incubated with different concentrations of Na_2_HPO_4_ for 48 h then their viability assessed via both MTT and live/dead assays. In brief, cells were seeded in 96 well plates at a concentration of 6 × 10^5^ cells/mL and volume of 100 µL. Cells were then incubated for 24 h at 37 °C and 5% CO_2_. After 24 h of incubation, Na_2_HPO_4_ was added to cells at variable concentrations (5, 10, 20, 40, 80, 100 and 120 mM) while maintaining a total volume of 200 µL for each sample. Cells with 200 µL media and no Na_2_HPO_4_ were kept as a control. Cells were then incubated for an additional 48 h. After 48 h, MTT or live/dead (Thermo Fisher Scientific, Waltham, Massachusetts, USA) were added to cells. Cells treated with MTT were subsequently incubated for extra 4 h. After 4 h, the absorbance of each sample was measured and subsequent cellular viability determined. For cells treated with live/dead, cells were incubated for 15 min and then imaged under a fluorescence microscope (Zeiss AxioCam HRm, Carl Zeiss, Oberkochen, Germany) at 10× magnification where live cells were stained green by Calcein AM and dead cells were stained red by ethidium homodimer-1. Percent cell viability was calculated as: Percentage cell viability = (number of live cells (green))/(number of live cells (green) + number of dead cells (red)) × 100. Cell counting was performed via ImageJ analysis software^44,45^.

To confirm that any observed effects are due to phosphate and not to osmotic pressure or to sodium in Na_2_HPO_4_, MTT assay was performed to quantify the toxicity of sodium and the effect of osmotic pressure. In particular, cells were incubated with 20 mM, 40 mM, 60 mM sucrose to examine the effect of osmotic pressure and with 20 mM, 40 mM NaCl and 20 mM sodium bicarbonate to examine effect of sodium.

To determine the mechanism of cell death induced by Na_2_HPO_4_ on MDA-MB-231 cells, cells were incubated with Na_2_HPO_4_ for 48 h and then treated with Apoptosis/Necrosis dyes (abcam, Cambridge, UK) following the manufacturer’s recommendations. Cells were then imaged using a fluorescence microscope. The images of fluorescent cells were taken at a specific z-plane and the background noise was removed during image analysis via “Noise Tolerance” option in ImageJ.

### Measurement of pH

In order to investigate the possible ways in which phosphate influences MDA-MB-231 and THP-1 cell survival, the pH of the resultant solutions (after adding different phosphate compounds and after adding different concentrations of Na_2_HPO_4_ and NaH_2_PO_4_) was measured using a pH meter (ORION STAR A211, Thermo Scientific).

### Tolerance of MDA-MB-231 and THP-1 cells to different pH levels

To study the tolerance of MDA-MB-231 and THP-1 cells to different pH levels, cells were incubated for 48 h in media having pH levels ranging from 5 to 9 then their viability was assessed using MTT assay. In brief, MDA-MB-231 and THP-1 cells were seeded in 96 well plates at a concentration of 6 × 10^5^ cells/mL and incubated for 24 h at 37 °C and 5% CO_2_. After 24 h of incubation, the old media was replaced by new media having its pH adjusted by HCl (Sigma-Aldrich) or NaOH (Sigma-Aldrich) to obtain a specific pH; either 5, 6, 8 or 9. Media without any addition of acid or base was kept as a control (pH 7.2). The cells were incubated for 48 h. After 48 h, MTT was added to cells and their viability was quantified.

### Cytotoxicity of sodium phosphate dibasic at physiological pH

To study the toxicity of Na_2_HPO_4_ on MDA-MB-231 and THP-1 cells at physiological pH, cells were incubated for 48 h with 20 mM sodium phosphate dibasic (Na_2_HPO_4_) solutions having their pH adjusted to 7.4 then their viability was assessed via MTT assay. In brief, cells were seeded in 96 well plates at a concentration of 6 × 10^5^ cells/mL and volume of 100 µL. Cells were then incubated for 24 h at 37 °C and 5% CO_2_. After 24 h of incubation, 20 mM Na_2_HPO_4_ at pH 8.1 and at pH 7.4 were added to the cells while maintaining a total volume of 200 µL for each sample. Two controls were maintained for this experiment: cells kept in 200 µL media at pH 7.4 (negative control) and cells kept in 200 µL media having its pH adjusted to 8 or 8.5 by NaOH (Sigma-Aldrich) (positive control). The cells were incubated for 48 h. After 48 h, MTT was added to cells and their viability was quantified.

### Data analysis

All experiments were done in triplicates with the sample number in each replicate being 3. The data is presented as mean ± standard error. Student t-test was performed to determine significance. P < 0.05 was regarded as significant (*), P < 0.01 was regarded as highly significant (**) and P < 0.001 was regarded as very highly significant (***).

## Supplementary Information

Supplementary Figures.
