# Supplementary material for: Hydrogen phosphate selectively induces MDA MB 231 triple negative breast cancer cell death in vitro
Source: Sci Rep. 2022 Mar 29;12:5333. doi: 10.1038/s41598-022-09299-2 (PMC8964734; doi:10.1038/s41598-022-09299-2)
Supplement: Supplementary file 1 — Supplementary Figures. [file 41598_2022_9299_MOESM1_ESM.docx]

Supplementary Figure S1


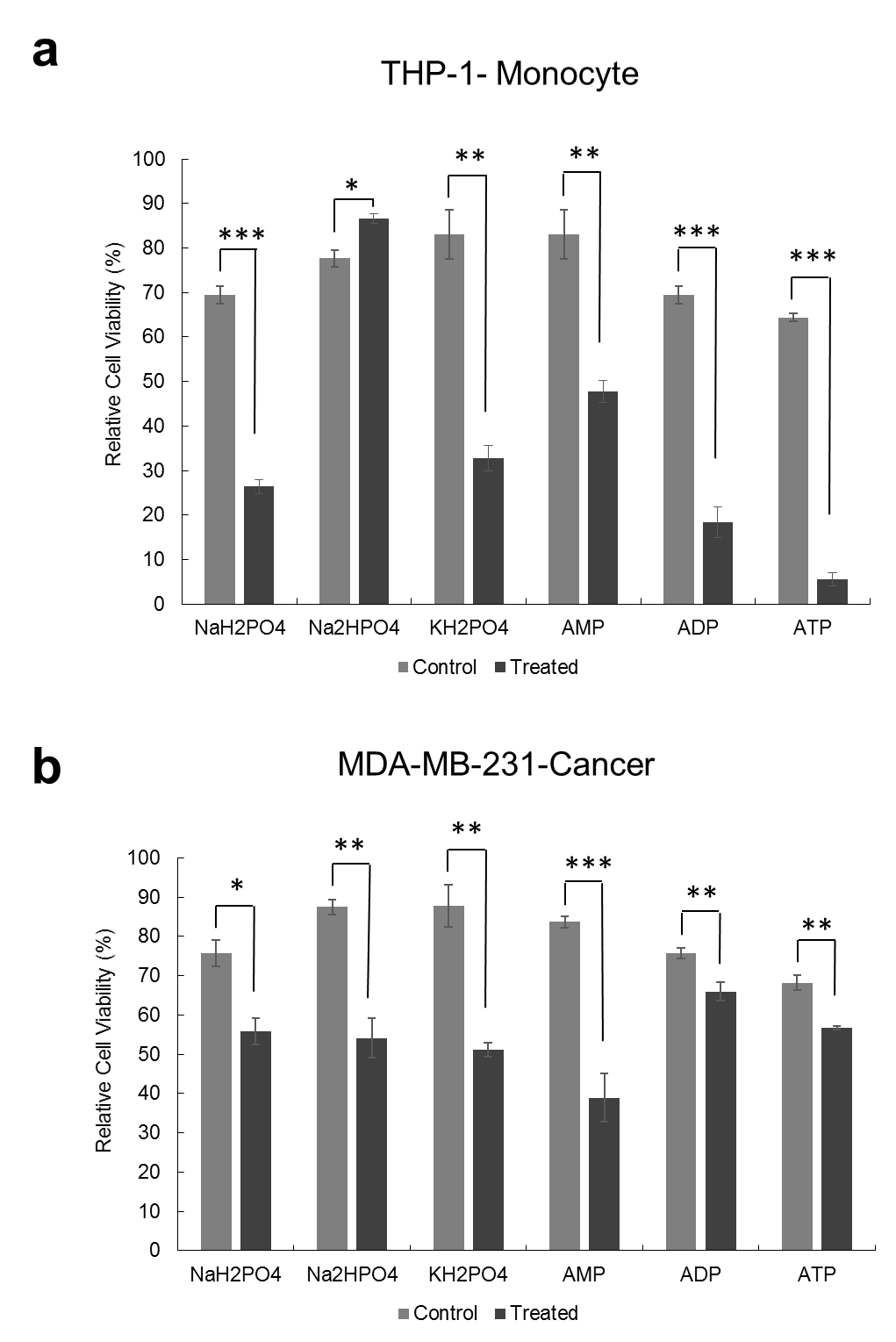


**Figure S1**: Toxicity of different phosphate compounds to (a) THP-1, human monocyte cell line, and (b) MDA-MB-231, human breast cancer cell line, evaluated after 48 hours of incubation with 20 mM of phosphate compound and compared to controls at suitable pH (i.e. to cells incubated in media with no phosphate compound but having their media pH adjusted by either HCl or NaOH to a level nearly equal to that containing phosphate compound).

Supplementary Figure S2

**
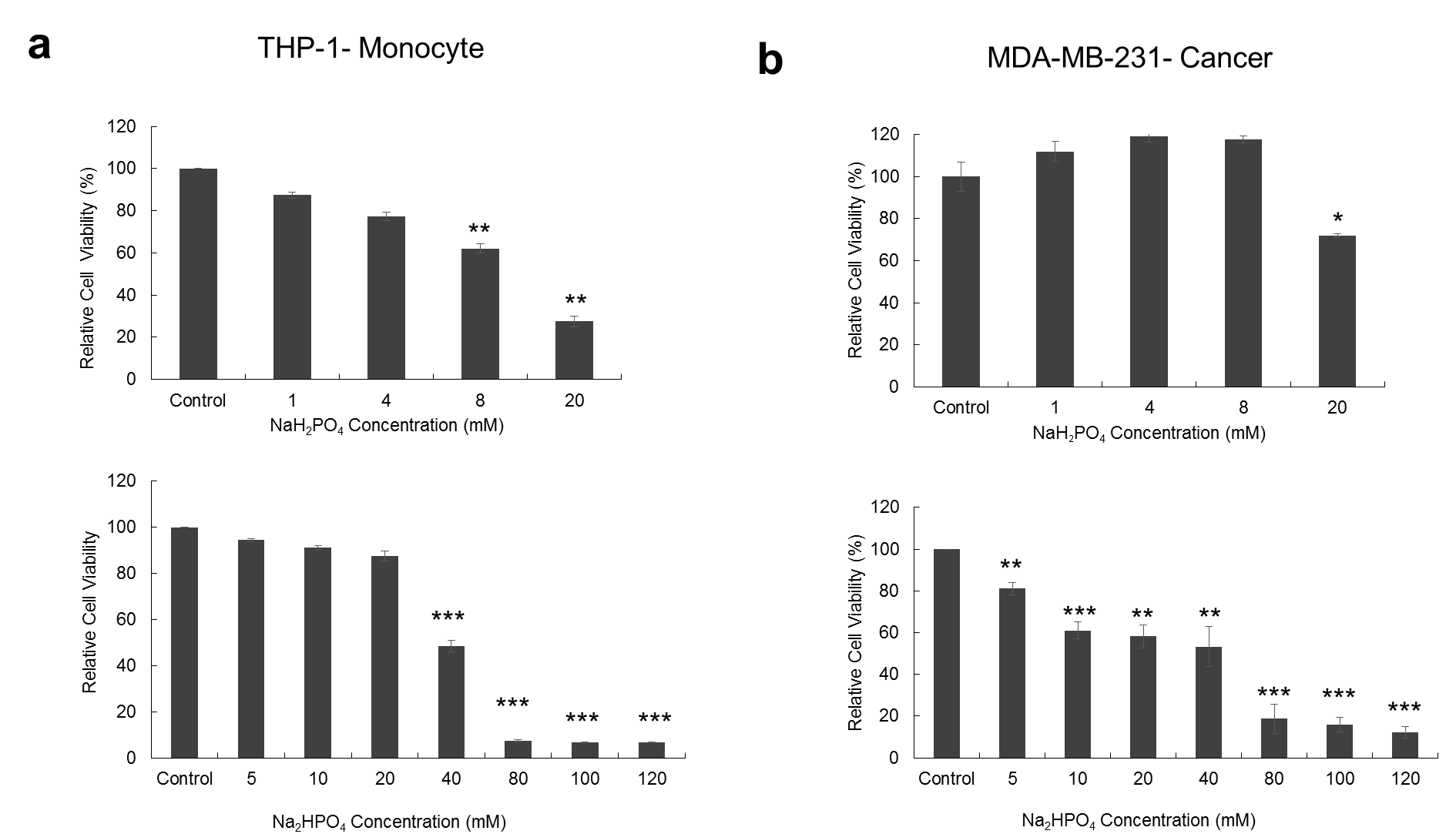
**

**Figure S2**: Toxicity of Sodium Phosphate Monobasic (NaH_2_PO_4_) and Sodium Phosphate Dibasic (Na_2_HPO_4_) to (a) THP-1 and (b) MDA-MB-231 cells assessed via MTT assay. At low concentrations (<10 mM), sodium phosphate monobasic is more toxic to THP-1 than it is to MDA-MB-231 indicating that it might not be a suitable therapeutic for treatment of cancer. However, at low concentrations (<10 mM), sodium phosphate dibasic is more toxic to MDA-MB-231 than it is to THP-1 indicating the therapeutic potential of this compound for treatment of cancer.
